# The Usefulness of a Preoperative Nomogram for Predicting the Probability of Conversion from Laparoscopic to Open Distal Pancreatectomy: A Single-Center Experience

**DOI:** 10.1007/s00268-020-05806-6

**Published:** 2020-10-15

**Authors:** Riccardo Casadei, Claudio Ricci, Carlo Ingaldi, Laura Alberici, Maria Chiara Vaccaro, Elisa Galasso, Francesco Minni

**Affiliations:** Department of Internal Medicine and Surgery (DIMEC), S.Orsola-Malpighi Hospital, Alma Mater Studiorum-University of Bologna, Via Massarenti n.9, 40138 Bologna, Italy

## Abstract

**Background:**

Laparoscopic distal pancreatectomy (LDP) represents a challenging procedure with a high conversion rate. A nomogram is a simple statistical predictive tool which is superior to risk groups. The aim of this study was to develop and validate a preoperative nomogram for predicting the probability of conversion from laparoscopic to open distal pancreatectomy.

**Methods:**

This is a retrospective study of 100 consecutive patients who underwent LDP. For each patient demographic, pre-intra- and postoperative data were collected. Univariate and multivariate analyses were carried out to identify the factors significantly influencing the conversion rate. The effect of each factor was weighted using the beta coefficient (*β*), and a nomogram was built. Finally, a logistic regression between the score and the conversion rate was carried out to calibrate the nomogram.

**Results:**

The conversion rate was 19.0%. At multivariate analysis, female (*β* =  − 1.8 ± 0.9; *P* = 0.047) and tail location of the tumor (*β* =  − 2.1 ± 1.1; *P* = 0.050) were significantly related to a low probability of conversion. Body mass index (BMI) (*β* = 0.2 ± 0.1; *P* = 0.011) and subtotal pancreatectomy (*β* = 2.4 ± 0.9; *P* = 0.006) were factors independently related to a high probability of conversion. The nomogram constructed had a minimum value of 4 and a maximum value of 18 points. The probability of conversion increased significantly starting from a minimum score of 6 points (*P* = 0.029; conversion probability 14.4%; 95%CI, 1.5–27.3%) up to 16 (*P* = 0.048; 27.8%; 95%CI, 0.2–48.7%).

**Conclusion:**

The nomogram proposed could serve as an effective preoperative tool capable of assessing the probability of conversion, allowing to take reliable decisions regarding indications and adequate stepwise training program of LDP.

## Introduction

Analysis from the American College of Surgeons National Surgical Quality Improvement Program (ACS-NSQIP) and the hepato-pancreato-biliary (HPB) collaborative has shown that 44.5% of distal pancreatectomies (DPs) are performed using a minimally invasive approach [1]. These data confirmed the safety, feasibility and efficacy of laparoscopic distal pancreatectomy (LDP), at least comparable to those obtained with the open distal pancreatectomy (ODP) as reported by several studies [2–8]. However, LDP represents a challenging procedure with different degrees of technical complexity, mainly related to patients’ habitus, tumor-related factors and the surgeon’s learning curve, which can influence the probability of conversion. Different scoring systems were proposed to recognize the different degrees of complexity of LDP, but they usually considered also intra-operative factors and did not result as being reliable in preoperatively predicting the probability of conversion [9, 10]. In addition, only a few articles have identified the preoperative risk factors predictive of conversion [11–13]. In 2018, the authors [13] identified some risk factors capable of increasing the odds of conversion; however, the conversion probability rate was not assessed for each procedure. A nomogram is a simple statistical predictive tool used as an algorithm to predict the probability of a given outcome [14]. Its construction is based on factors having different weights, capable of influencing the outcome of the procedure considered. Predictions based on a nomogram are more accurate than those based on clinical judgment and experience, and are superior to risk groups as well [14]. Thus, the aim of the present study was to develop and validate a preoperative nomogram for predicting the probability of conversion from laparoscopic to open distal pancreatectomy, assessing its usefulness and reliability in the indication and training for LDP.

## Materials and methods

### Study design

This was a retrospective study based on a prospectively maintained database of LDPs from January 2004 to 2020; it was approved by Ethical Committee of S. Orsola-Malpighi Hospital (642017 UOss) with patient informed consent. The following data were collected for each patient: gender, age, co-morbidities, American Society of Anesthesiologists (ASA) score, body mass index (BMI), previous abdominal surgery, tumor size and site, type of lesion (solid/cystic), suspicion of malignancy, completed learning curve, cumulative procedures for the surgeon, type of pancreatic resection (left pancreatectomy or subtotal pancreatectomy), spleen resection (yes or no), parenchymal thickness at the resection line, expected texture of the pancreatic parenchyma (soft/hard), extended resection (extension of procedure to neighboring organs), pathological diagnosis (pancreatic ductal adenocarcinoma—PDAC vs. non-PDAC) and conversion rate. The postoperative data (mortality and morbidity, pancreatic fistula, post-pancreatectomy hemorrhage, reoperation rate and length of hospital stay) were also reported, but not included in the analysis.

### Terminology and definition

The surgical technique was previously reported [13]. For each surgeon, the learning curve was considered completed after 17 procedures, as previously reported [15], and all 3 surgeons who performed the LDPs (RC, CR and FM) had completed their learning curve. Open conversion was defined as any laparotomy or hand assistance for other reasons than trocar placement or specimen extraction. When the tumor was in the body of the pancreas, it meant that the tumor was located between the left border of the portal vein and the left border of aorta; when the tumor was located in the pancreatic tail, it meant that the tumor was distal to the left border of the aorta. Left pancreatectomy and subtotal pancreatectomy were defined as the transection of the pancreas on the left and on the right of the portal vein, respectively. In a subtotal pancreatectomy, the resection line was at the level of the portal vein requiring a tunneling procedure, while in a left pancreatectomy, the tunneling procedure was not required. An extended procedure was defined as a surgical resection involving other neighboring organs in addition to the pancreas. Postoperative mortality was defined as the number of deaths occurring during hospitalization or within 90 days after surgery. Postoperative morbidity included all complications following surgery up to the day of discharge according to the Clavien–Dindo classification [16]. A postoperative pancreatic fistula (POPF) was defined according to the 2016 definition proposed by the International Study Group of Pancreatic Fistula (ISGPF) [17]. Post-pancreatectomy hemorrhage (PPH) was defined as intra-abdominal or intestinal bleeding according to the criteria of the International Study Group of Pancreatic Surgery (ISGPS) [18]. Reoperation was defined as any surgical procedure performed in the first 30 postoperative days or before discharge from the hospital. Length of hospital stay (LOS) was calculated as the interval from the day of surgery to the date of discharge.

### Statistical analysis

All the categorical variables were described as frequencies and percentages, while the continuous variables were reported as medians and interquartile ranges. The analysis was carried out in three steps. First, univariate and multivariate analyses were carried out in order to identify all the factors significantly influencing the conversion rate. The univariate analysis was carried out using the Fisher’s exact test, Pearson’s Chi-square test and the Student’s t test for dichotomic, ordinal and continuous variables, respectively. The multivariate analysis was carried out using stepwise backward logistic regression. The effect of each factor was described using the beta coefficient (*β*) with standard error (SE). When the *β* value was >0, the probability of conversion increased, while when the *β* value was <0 the probability decreased. A two-sided *P* value <0.05 was considered statistically significant. Second, the *β* values were used to build a nomogram using a dedicate algorithm [19]. Only the factors remaining in the last step of the backward stepwise multivariate analysis (*P* value <0.050) were included in the nomogram, and the *β* values were converted into integers number (from 0 to 10 points) for each factor included. Moreover, we calculated the area under the curve (AUC), sensitivity, specificity, false positive and negative rates, and positive and negative predictive values of the nomogram. Finally, a score was generated which was the sum of all the points allocated to each factor. Third, an internal validation of the score was obtained calculating the ability of the score in predicting the probability of conversion. For this purpose, logistic regression between the score and the conversion rate was carried out. The results were reported for each score point as post-estimation probability of conversion within 95% confidence interval (95% CI). A two-sided *P* value <0.05 indicated a significant increase in probability for each point with respect to the previous value.

## Results

**Fig. 1:**
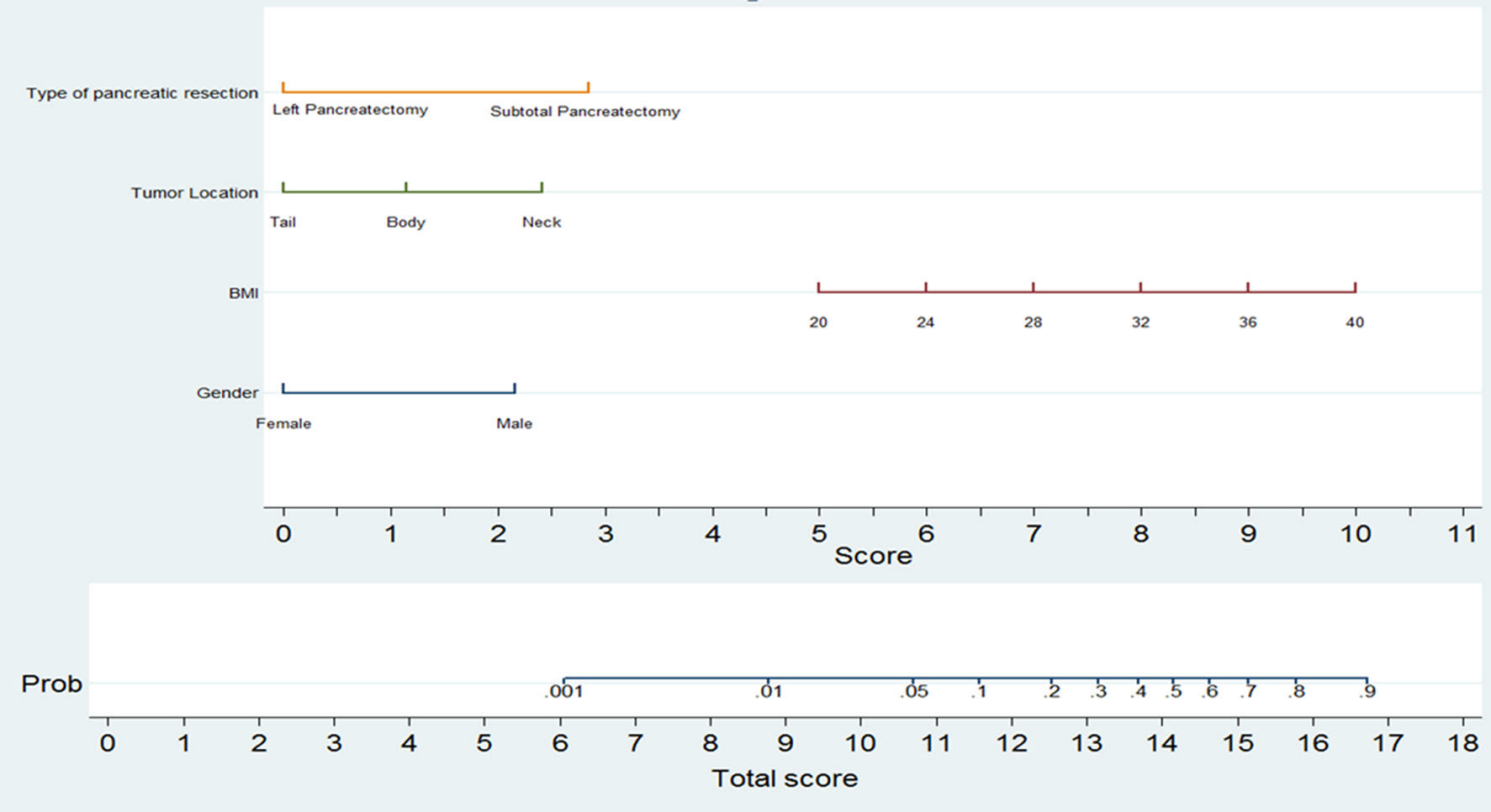
The preoperative nomogram is plotted utilizing 4 variables, resulted as independent factors at multivariate analysis, and weighted basing on *β* coefficient. The resulting score system had a minimum value of 4 and a maximum value of 18 points

**Fig. 2:**
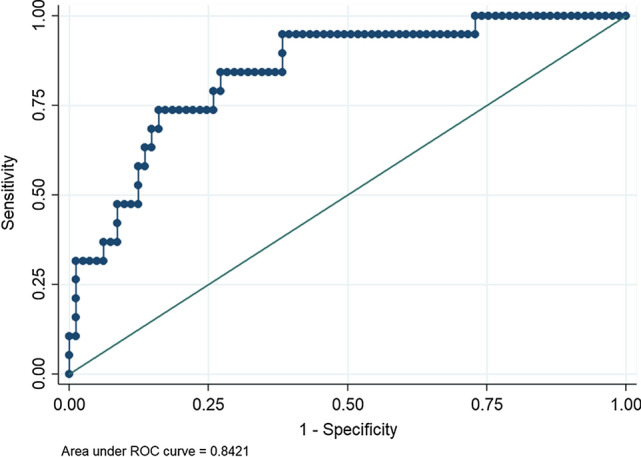
Area under the curve (AUC) of the nomogram. The AUC is 0.8421, and the patients properly classified are 82%. Sensitivity and specificity were 31.6 and 93.8%, respectively

**Fig. 3:**
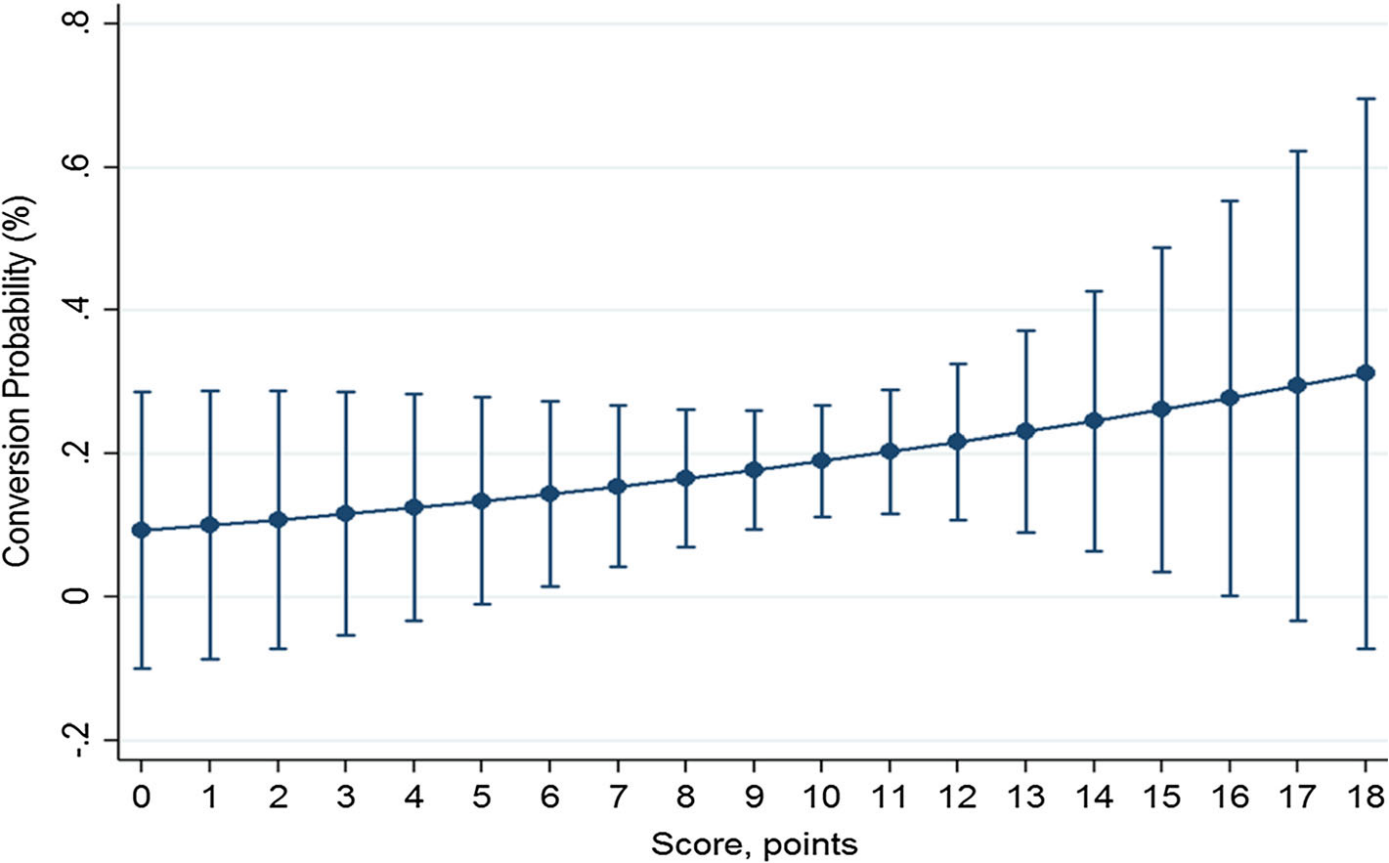
Post-estimation data are plotted in the calibration curve. It showed that the conversion probability ranges from 9.1 to 31.2% and increases significantly from 6 to 16 points

From January 2004 to 2020, a total of 100 LDP were carried out. Of the latter, 19 (19.0%) required a conversion from laparoscopic to open distal pancreatectomy, while 81 (81.0%) successfully completed the laparoscopic distal pancreatectomy. Descriptive data regarding the characteristics of the patients and postoperative outcomes are summarized in Tables 1 and 2. Univariate and multivariate analyses of the factors influencing the conversion from LDP to ODP are reported in Table 3. At univariate analysis, a high BMI (*P* = 0.017), subtotal pancreatectomy (*P* = 0.002) and the presence of co-morbidities (*P* = 0.035) were significantly related to open conversion. A tumor located in the neck or body of the pancreas (*P* = 0.055) and a diagnosis of PDAC (*P* = 0.086) showed a trend in relation to open conversion. At multivariate analysis, female (*β* = − 1.8 ± 0.9; *P* = 0.047) and a tumor located in the tail (*β* = − 2.1 ± 1.1; *P* = 0.050), were protective factors regarding an open conversion (negative *β*-coefficient). Conversely, a subtotal pancreatectomy (*β* = 2.4 ± 0.9; *P* = 0.006) and a high BMI (*β* = 0.2 ± 0.1; *P* = 0.011) were factors independently related to an increased probability of conversion. Utilizing the independent factors, a nomogram was plotted as shown in Fig. 1. Based on the *β* coefficient, the nomogram allowed calculating the following scores: subtotal pancreatectomy 3 points; neck location of the lesion 3 points; body location 1 point; and male gender 2 points. The BMI was given: 4 points for values <20 kg/m^2^; 5 points for values ≥20 and <24; 6 points for values ≥24 and <28; 7 points for values ≥28 and <32; 8 points for values ≥32 and <36; 9 points for values ≥36 and <40; and 10 points for values ≥40. Assuming that the patients at risk of conversion were those patients with a risk of conversion >50%, the AUC was 0.842, and the patients properly classified were 82% (Fig. 2). Sensitivity, specificity, positive predictive value and negative predictive value were 31.6, 93.8, 54.6 and 85.4%, respectively. The false positive rate and the false negative rate were 45.5 and 14%, respectively. The resulting score system had a minimum value of 4 and a maximum value of 18 points. To validate the nomogram, logistic regression was carried out, which showed that the probability of conversion increased significantly starting from a minimum score of 6 points (*P* = 0.029): For each single-point increase in the score, the probability of conversion increased significantly (*P* <0.05); however, for points >16 (*P* = 0.048), the increase in probability was not statistically significant. The probability of conversion remained very low until a score of 6 points was reached (14.4, 95% CI 1.5–27.3%); it reached the highest significant probability at 27.8% (95%CI: 0.2–48.7%) with a score of 16 points. Post-estimation data are shown in Table 4 and plotted in Fig. 3 (calibration curve).

**Table 1:** Descriptive data of 100 patients who underwent laparoscopic distal pancreatectomy

Variable	N (%) or median (IQR)
*Gender*	
Female	59 (59.0)
Male	41 (41.0)
*Age (years)*	60 (53–73)
Co-morbidity	
None	39 (39.0)
One or more	61 (61.0)
*ASA score*	
I	5 (5.0)
II	48 (48.0)
III	47 (47.0)
BMI (Kg/m^2^; median; range)	25.2 (22.7–28.5)
*Previous abdominal surgery*	
No	45 (45.0)
Laparoscopic	6 ( 6.0)
Open	49 (49.0)
Tumor size (mm; median; range)	25 (15–40)
*Tumor site*	
Neck	19 (19.0)
Body	36 (36.0)
Tai	45 (45.0)
*Type of lesion*	
Solid neoplasm	42 (42.0)
Cystic neoplasm	58 (58.0)
*Suspicion of malignancy*	
No	41 (41.0)
Yes	59 (59.0)
*Completed learning curve (>17 procedures)*	
No	43 (43.0)
Yes	57 (57.0)
Cumulative procedures per surgeon (median; range)	20 (7.5–32)
*Spleen resection*	
No	21 (21.0)
Yes	79 (79.0)
*Type of pancreatic resection*	
Left pancreatectomy	82 (82.0)
Subtotal pancreatectomy	18 (18.0)
Parenchymal thickness at the resection line (mm; median; range)	16.5 (12–22)
*Texture of the pancreatic parenchyma*	
Soft	64 (64.0)
Hard	36 (36.0)
*Extended resection*	
No	95 (95.0)
Yes	5 (5.0)
*Pathological diagnosis*	
PDAC	14 (14.0)
Non-PDAC	86 (86.0)
NET	36 (36.0)
IPMN	17 (17.0)
MCN	14 (14.0)
SCN	7 (7.0)
Others	12 (12.0)
*Conversion rate*	
No	81 (81.0)
Yes	19 (19.0)

*IQR* Interquartile range, *ASA* American Society of Anesthesiologists, *BMI* body mass index, *PDAC* pancreatic ductal adenocarcinoma, *NET* neuroendocrine tumor, *IPMN* intra-ductal papillary mucinous neoplasm, *MCN* mucinous cystic neoplasm, *SCN* serous cystic neoplasm

**Table 2:** Postoperative course in the 100 patients who underwent laparoscopic distal pancreatectomy

Postoperative course	N (%)
Mortality	0 (100)
*Morbidity according to the C-D*	
No	48 (48.0)
1	13 (13.0)
2	23 (23.0)
3a	5 (5.0)
3b	5 (5.0)
4	1 (1.0)
*POPF according 2016 ISGPF definition*	
No	71 (71.0)
Grade B	27 (27.0)
Grade C	2 (2.0)
*PPH according ISGPS*	
No	91 (91.0)
Grade A	2 (2.0)
Grade B	5 (5.0)
Grade C	2 (2.0)
*Reoperation*	
No	95 (95.0)
Yes	5 (5.0)
LOS (days, median, range)	9.5 (8–12

*C*–*D* Clavien–Dindo classification system, *POPF* postoperative pancreatic fistula, *ISGPF* International Study Group of Pancreatic Fistula, *PPH* post-pancreatectomy hemorrhage, *ISGPS* International Study Group of Pancreatic Surgery, *LOS* length of stay

**Table 3:** Factors influencing the conversion in the 100 patients who underwent laparoscopic distal pancreatectomy

Variables	Univariate				Multivariate	
	LDP	CLDP	*P* value	*β* ± SE	*P* value	Exclusion step
Gender					0.047	Last
Male (%)	50 (84.7)	9 (15.3)	0.304	Referent		
Female (%)	31 (75.6)	10 (24.4)		−1.8 ± 0.9		
Age (years; median; range)	60 (54–70)	66 (51–75)	0.473	−0.1 ± 0.1	0.228	10th
Co-morbidity			0.035		0.606	
None (%)	36 (92.3)	3 (7.7)		Referent		5th
One or more (%)	45 (73.7)	16 (26.3)		1.3 ± 2.5		
ASA						
I (%)	5 (100.0)	0 (0.0)	0.214	−1.1 ± 1.7	0.502	
II (%)	41 (85.4)	7 (14.6)		*	*	7th
III (%)	35 (74.5)	12 (25.5)		*	*	
BMI (Kg/m^2^; median; range)	24.9 (22.8–27.9)	27.6 (22.7–33.3)	0.017	0.2 ± 0.1	0.011	Last
Previous abdominal surgery						
No (%)	35 (77.8)	10 (22.2)		Referent		9th
Laparoscopic (%)	3 (50.0)	3 (50.0)	0.064	1.9 ± 2.2	0.388	
Open (%)	43 (87.8)	6 (12.2)		−0.2 ± 1.4	0.863	
Size of lesions (mm; median; range)	25 (16–40)	10 (23–35)	0.661	0.1 ± 0.1	0.649	6th
Site of lesion						Last
Neck (%)	13 (68.4)	6 (31.6)	0.055	Referent		
Body (%)	27 (75.0)	9 (25.0)		−1.1 ± 0.9	0.258	
Tail (%)	41 (91.1)	4 (8.9)		−2.1 ± 1.1	0.050	
Type of lesion			0.038		0.532	8th
Solid neoplasm (%)	30 (71.4)	12 (28.6)		Referent		
Cystic neoplasm (%)	51 (87.9)	7 (12.1)		−0.7 ± 1.2		
Suspicion of malignancy			0.531		0.429	12th
No (%)	49 (83.1)	10 (16.9)		Referent		
Yes (%)	51 (87.9)	7 (12.1)		0.8 ± 1.1		
PDAC			0.086		0.232	11th
No (%)	72 (83.7)	14 (16.3)		Referent		
Yes (%)	9 (64.3)	5 (36.7)		2.5 ± 2.1		
Learning curve			0.547		0.882	2nd
No (%)	36 (83.7)	7 (16.3)		Referent		
Yes (%)	45 (78.9)	12 (21.1)		0.6 ± 3.9		
Cumulative procedures per surgeon (median; range)	19 (7–32)	20 (11–33)	0.849	−0.1 ± 0.1	0.905	3rd
Spleen resection			0.213			1st
No (%)	19 (90.5)	2 (9.5)		Referent	0.987	
Yes (%)	62 (78.5)	17 (21.5)		−0.4 ± 3.1		
Type of pancreatic resection			0.002		0.006	Last
Left pancreatectomy (%)	71 (86.6)	11 (13.4)		Referent		
Subtotal pancreatectomy (%)	10 (55.6)	8 (44.4)		2.4 ± 0.9		
Parenchymal thickness at resection line (mm; median; range)	16 (12–22)	19 (12–22)	0.833	−0.1 ± 0.2	0.863	4th
Texture of the pancreatic parenchyma			0.328		0.148	13th
Soft (%)	50 (78.1)	14 (21.9)		Referent		
Hard (%)	31 (86.1)	5 (13.9)		−1.5 ± 1.1		
Extended resection			0.219		0.205	14th
No (%)	78 (82.1)	17 (17.9)		Referent		
Yes (%)	3 (60.0)	2 (40.0)		−2.5 ± 1.9		

*ASA* American Society of Anesthesiologists, *BMI* body mass index, *PDAC* pancreatic ductal adenocarcinoma, *LDP* laparoscopic distal pancreatectomy, *CLDP* converted laparoscopic distal pancreatectomy, *β* beta coefficient of logistic regression

**Table 4:** Post-estimation value scores

Score, points	Conversion probability in % (95% CI)	*P* value*
0	Referent	Referent
1	9.9 (0–28.7)	0.296
2	10.7 (0–28.7)	0.240
3	11.6 (0–28.6)	0.181
4	12.5 (0–28.3)	0.123
5	13.4 (0–27.8)	0.069
6	14.4 (1.5–27.3)	0.029
7	15.4 (4.2–26.7)	0.007
8	16.5 (7.1–26.1)	0.001
9	17.7 (9.4–25.9)	<0.001
10	18.9 (11.2–26.7)	<0.001
11	20.2 (11.6–28.8)	<0.001
12	21.6 (10.8–32.5)	<0.001
13	23.1 (9.1–37.1)	<0.001
14	24.5 (6.5–42.7)	0.008
15	26.1 (3.5–48.7)	0.024
16	27.8 (0.2–48.7)	0.048
17	29.4 (0–62.2)	0.079
18	31.2 (0–69.6)	0.112

*ASA* American Society of Anesthesiologists, *BMI* body mass index, *PDAC* pancreatic ductal adenocarcinoma

^*^Logistic regression between score and conversion rate: *P* < 0.05 means that the probability related to the score value is statistically significant as compared to the previous score value

## Discussion

In the first multicenter randomized controlled trial of minimally invasive versus open distal pancreatectomy, de Rooij T et al. [20] concluded that: “the treatment of choice for patients with left-sided pancreatic tumors is minimally invasive distal pancreatectomy, from both a clinical and quality-of-life points of view, when performed by appropriately trained surgeons.” Nevertheless, the ACS-NSQIP [1] reported that more than 50% of patients underwent open distal pancreatectomy, and the conversion rate reported in the literature regarding LDP was high, ranging from 6.5 to 27.3% [21, 22]. These data confirmed that LDP is a challenging procedure and produces some questions regarding when and why of selecting the minimally invasive approach for patients with left-sided pancreatic tumors. In this context, a preoperative tool capable of predicting the probability of conversion from LDP to ODP would be very useful for patient selection and adequate completion of the learning curve. In the authors’ previous article [13], some risk factors were identified to be predictive of the major difficulty of the laparoscopic procedure. These factors seemed to be useful in differentiating the easy laparoscopic procedures, at low risk of conversion, from the difficult procedures, at high risk of conversion. However, in the previous study, the assessment of the probability of conversion from LDP to ODP for each procedure was lacking. To the authors’ knowledge, the present is the first study which used a preoperative nomogram for this aim. In this study, the nomogram was built considering four variables, which were significantly related to conversion at multivariate analysis and were weighted differently according to the beta coefficient: type of pancreatic resection (left pancreatectomy versus subtotal pancreatectomy), tumor location (tail, body, neck), BMI and gender. The AUC showed that the nomogram was capable to properly classified the major part of the patients (82%). In particular, patients at risk of conversion were quite always identified (specificity, 93.8%). This datum means that the nomogram is very effective. The scoring system resulted in a minimum value of 4 and a maximum value of 18 points. The probability of conversion increased significantly, for each point, starting from a minimum score of 6 points (conversion probability 14.4%; 95% CI 1.5–27.3; *P* = 0.029) up to a score of 16 points (conversion probability 27.8%; 95% CI 0.2–48.7; *P* = 0.048). This datum points out that the difficulty of the laparoscopic approach increases significantly from each point in the interval between 6 and 16 points, thus involving some considerations.

Below a score of 6 points, the probability of conversion was low (from 9.9 to 13.4%) and there were no significant differences from 0 to 5 points. This meant that the laparoscopic procedure seemed easy, in these cases, because the factors related to the patient’ habitus and disease characteristics did not emphasize the technical limitation of the laparoscopic approach which was not technically difficult. In this scenario, it was reasonable to suggest that the laparoscopic approach was clearly indicated and represented the treatment of choice for the pancreatic lesion. In addition, it could be performed at an early stage of the learning curve.

From 6 to 16 points, significant differences in the probability of conversion were reported for each point (from 14.4 to 27.8%). Thus, in this interval, the difficulty of the laparoscopic approach increased significantly for each point. In these cases, the selection of the patients had to be carefully assessed taking into account the factors related to both patient and disease. Moreover, it should be noted that the technical limitation of the laparoscopic approach as well as the limited range of motion, two-dimensional visualization and difficulty in controlling large blood vessels could be more evident in these cases than in those having a score of <6 points. In this setting, the laparoscopic procedure has to be performed by an experienced laparoscopic surgeon who has completed his/her learning curve. Finally, in those cases with a very high probability of conversion (score 16 points, 27.8% conversion probability), it could be hypothesized that the laparoscopic procedure was very difficult and the decision to perform a minimally invasive or an open approach had to be assessed carefully. In the latter scenario, the technical limitation of the laparoscopic approach was pointed out even if performed by an experienced laparoscopic surgeon. The choice of the proper approach, minimally invasive or open, is difficult, and the open approach is sometimes preferred. In fact, it should be noted that recent studies have reported an increase in morbidity, 30-day mortality and length of stay in patients who underwent conversion from LDP to ODP [11, 23]. On the other hand, some authors [22] have recently reported that elective conversion was associated with outcomes similar to ODP, while, on the contrary, only emergency conversion, mainly due to bleeding, was related to worse outcomes. In summary, in cases with a high probability of conversion, the indication for LDP should be carefully assessed by an experienced laparoscopic surgeon who can avoid an emergency conversion, can take advantage of minimally invasive approach for easier dissection and enhanced visualization and can perform early conversion when unexpected intra-operative findings are encountered.

The present study has several limitations. The nomogram was constructed using retrospective data from a prospective single-center database, over a long period of time, and this could have led to the risk of potential bias. Finally, an external validation, using another series from another center, is suggested in order to validate the efficacy of the nomogram.

In conclusion, the present study, despite its limitations, confirmed that LDP is a challenging procedure with a high conversion rate (19.0%). The construction of a preoperative nomogram, based on factors related to patient habitus and disease characteristics, to assess the probability of conversion from LDP to ODP seemed to represent a useful and reliable preoperative tool for each patient. It seemed to properly suggest when the LDP was the procedure of choice, when it had a high risk of conversion or when the open approach was preferable. The nomogram also seemed to be useful in assessing how to perform an adequate stepwise training program for each surgeon. Additional studies are needed for the external validation of the proposed nomogram with the aim of becoming well acquainted with its usefulness and reliability.

## References

[1] Røsok BI, de Rooij T, van Hilst J (2017). Minimally invasive distal pancreatectomy. HPB.

[2] Zureikat AH, Borrebach J, Pitt HA (2017). Minimally invasive hepatopancreatobiliary surgery in North America: an ACS-NSQIP analysis of predictors of conversion for laparoscopic and robotic pancreatectomy and hepatectomy. HPB.

[3] Venkat R, Edil BH, Schulick RD (2012). Laparoscopic distal pancreatectomy is associated with significantly less overall morbidity compared to the open technique: a systematic review and meta-analysis. Ann Surg.

[4] Mehrabi A, Hafezi M, Arvin J (2015). A systematic review and meta-analysis of laparoscopic versus open distal pancreatectomy for benign and malignant lesions of the pancreas: it’s time to randomize. Surgery.

[5] Ricci C, Casadei R, Lazzarini E (2014). Laparoscopic distal pancreatectomy in Italy: a systematic review and meta-analysis. Hepatobiliary Pancreat Dis Int.

[6] Ricci C, Casadei R, Taffurelli G (2015). Laparoscopic versus open distal pancreatectomy for ductal adenocarcinoma: a systematic review and meta-analysis. J Gastrointest Surg.

[7] Ricci C, Casadei R, Taffurelli G (2016). Laparoscopic distal pancreatectomy: many meta-analyses, few certainties. Updates Surg.

[8] Casadei R, Ricci C, D'Ambra M (2010). Laparoscopic versus open distal pancreatectomy in pancreatic tumours: a case-control study. Updates Surg.

[9] Ohtsuka T, Ban D, Nakamura Y (2018). Difficulty scoring system in laparoscopic distal pancreatectomy. J Hepatobiliary Pancreat Sci.

[10] Goh BKP, Kabir T, Koh YX (2019). External validation of the Japanese difficulty scoring system for minimally-invasive distal pancreatectomies. Am J Surg.

[11] Goh BKP, Chan CY, Lee SY (2017). Factors associated with and consequences of open conversion after laparoscopic distal pancreatectomy: initial experience at a single institution. ANZ J Surg.

[12] Hua Y, Javed AA, Burkhart RA (2017). Preoperative risk factors for conversion and learning curve of minimally invasive distal pancreatectomy. Surgery.

[13] Casadei R, Ricci C, Pacilio CA (2018). Laparoscopic distal pancreatectomy: which factors are related to open conversion? Lessons learned from 68 consecutive procedures in a high-volume pancreatic center. Surg Endosc.

[14] Touijer K, Scardino PT (2009). Nomograms for staging, prognosis, and predicting treatment outcomes. Cancer.

[15] Ricci C, Casadei R, Buscemi S (2015). Laparoscopic distal pancreatectomy: what factors are related to the learning curve?. Surg Today.

[16] Casadei R, Ricci C, Pezzilli R (2011). Assessment of complications according to the Clavien-Dindo classification after distal pancreatectomy. JOP.

[17] Bassi C, Marchegiani G, Dervenis C (2017). The 2016 update of the International Study Group (ISGPS) definition and grading of postoperative pancreatic fistula: 11 years after. Surgery.

[18] Ricci C, Casadei R, Buscemi S (2012). Late postpancreatectomy hemorrhage after pancreaticoduodenectomy: is it possible to recognize risk factors?. JOP.

[19] Kattan MW, Eastham JA, Stapleton AMF (1998). A preoperative nomogram for disease recurrence following radical prostatectomy for prostate cancer. J Natl Cancer Inst.

[20] de Rooij T, van Hilst J, van Santvoort H (2019). Minimally Invasive Versus Open Distal Pancreatectomy (LEOPARD): a multicenter patient-blinded randomized controlled trial. Ann Surg.

[21] Abu Hilal M, Richardson JR, de Rooij T (2016). Laparoscopic radical ‘no-touch’ left pancreatosplenectomy for pancreatic ductal adenocarcinoma: technique and results. Surg Endosc.

[22] Lof S, Korrel M, van Hilst J (2019). Outcomes of elective and emergency conversion in minimally invasive distal pancreatectomy for pancreatic ductal adenocarcinoma: an international multicenter propensity score-matched study. Ann Surg.

[23] Nassour I, Wang SC, Porembka MR (2017). Conversion of minimally invasive distal pancreatectomy: predictors and outcomes. Ann Surg Oncol.

